# Training Design, Performance Analysis, and Talent Identification—A Systematic Review about the Most Relevant Variables through the Principal Component Analysis in Soccer, Basketball, and Rugby

**DOI:** 10.3390/ijerph18052642

**Published:** 2021-03-05

**Authors:** José Pino-Ortega, Daniel Rojas-Valverde, Carlos D. Gómez-Carmona, Markel Rico-González

**Affiliations:** 1 Department of Physical Activity and Sport Sciences, Faculty of Sports Sciences, International Excellence Campus “Mare Nostrum”, University of Murcia, 30720 San Javier, Spain; josepinoortega@um.es; 2 BIOVETMED & SPORTSCI Research Group, University of Murcia, 30100 Murcia, Spain; 3 Centro de Investigación y Diagnóstico en Salud y Deporte (CIDISAD), Escuela de Ciencias del Movimiento Humano y Calidad de Vida (CIEMHCAVI), Universidad Nacional, Heredia 86-3000, Costa Rica; 4 Research Group in Optimization of Training and Sports Performance (GOERD), Department of Didactics of Music, Plastic and Body Expression, Sports Science Faculty, University of Extremadura, 10071 Caceres, Spain; cdgomezcarmona@unex.es; 5 Departament of Physical Education and Sport, University of the Basque Country, UPV-EHU, Lasarte 71, 01007 Vitoria-Gasteiz, Spain

**Keywords:** team sport, exploratory factor analysis, PCA, big data, data mining

## Abstract

Since the accelerating development of technology applied to team sports and its subsequent high amount of information available, the need for data mining leads to the use of data reduction techniques such as Principal Component Analysis (PCA). This systematic review aims to identify determinant variables in soccer, basketball and rugby using exploratory factor analysis for, training design, performance analysis and talent identification. Three electronic databases (PubMed, Web of Science, SPORTDiscus) were systematically searched and 34 studies were finally included in the qualitative synthesis. Through PCA, data sets were reduced by 75.07%, and 3.9 ± 2.53 factors were retained that explained 80 ± 0.14% of the total variance. All team sports should be analyzed or trained based on the high level of aerobic capacity combined with adequate levels of power and strength to perform repeated high-intensity actions in a very short time, which differ between team sports. Accelerations and decelerations are mainly significant in soccer, jumps and landings are crucial in basketball, and impacts are primarily identified in rugby. Besides, from these team sports, primary information about different technical/tactical variables was extracted such as (a) soccer: occupied space, ball controls, passes, and shots; (b) basketball: throws, rebounds, and turnovers; or (c) rugby: possession game pace and team formation. Regarding talent identification, both anthropometrics and some physical capacity measures are relevant in soccer and basketball. Although overall, since these variables have been identified in different investigations, further studies should perform PCA on data sets that involve variables from different dimensions (technical, tactical, conditional).

## 1. Introduction

In the last decade, team sports have experienced an accelerating growth and evolution in technological developments (e.g., wearable, small, and inter-device connection), influencing the daily work from researchers to practitioners in the sports science area. Thanks to this development, new and specific tools have been created to use in team sports science and medicine that are safer, less invasive and with high validity and reliability [1,2]. The creation of these technological tools led to the development of different software to capture and analyze up to a thousand data per second in up to 400 variables after or in real-time from different dimensions (technical, tactical, conditional) [3]. 

The analysis and interpretation of a large amount of data derived from new technological devices in a short time represent a supreme challenge to the team sports’ scientists [4]. These data sets or combinations of data sets must be managed as big data due to its volume, complexity, variability; that requires special data management, processing and analysis [5]. The performance analysis in team sports involves the exploration of different types of variables (technical, tactical, conditional), being a challenge to the realization of a modeling process that allows the global understanding of the team behavior.

In the practical context, the assessment of training/competition workloads is necessary to design the exercise prescription (task design and performance analysis), as well as for talent identification. For this process, the identification of those variables which provide the most relevant information about players’ technical, tactical conditional and anthropometrical level/characteristics are very important for checking later the explaining performance in a separate step, which could be through like regression analysis, discriminant analysis or artificial neural networks, among others. Due to the large amount of information that needs to be reduced in team sports, different techniques for multivariate data analysis have been proposed [4]. For example, exploratory factor analysis (EFA) is commonly used in sports research to explain many measured variables using a smaller number of extracted factors [6,7,8]. These variables can then be used in the following analysis, such as *Cluster* or regression analysis, to provide a better explanation of some sports behaviors [9,10]. Despite the several factor extraction methods (e.g., maximum likelihood, alpha factoring, generalized least-products, unweighted least-squares, or principal axis factoring), Principal Component Analysis (PCA) has been considered an appropriate alternative when the purpose is to reduce a large amount of data into a reduced number of variables, especially in the sport context [11,12].

PCA has been considered a method of statistical analysis for data reduction to explain the most relevant variables of the players’ behavior in a number of different sports such as rugby [8,13], soccer [14], or basketball [15]. Biomechanical, physical, physiological and anthropometrical variables have been explored using PCA as a data reduction technique in the three sports [8,9,13,14]. In rugby and soccer, studies have focused their analysis on exploring which variables better explain the locomotion and kinematic behavior during training and official matches [8,13]. In contrast, its use in basketball has usually explored some technical and tactical variables [16,17]. Moreover, the PCA has been used to identify the most relevant variables for talent identification is young team sports players [18].

However, since each team sport has different characteristics such as space, time, or others, and it may be a reason for different players’ behavior, it is expected that different PCs were extracted in each of them. Also, in the same sport, the change of input variables, the phase of the season and other contextual variables may impact the factors obtained from the model. Due to these differences, the amount of data and the number of variables that could explain the total variance of each sport may vary. In this sense, some sports or different constraints in the same sport would need a larger number of variables and/or principal components to explain the total variance. The study of these potential differences and sport specifications could elevate the understanding of those variables and indicators to improve the knowledge of sports performance. Therefore, considering there is an increasing interest in using PCA in performance analysis as a data reduction technique; this systematic review aims to identify the key indicators of three team sports (soccer, basketball, and rugby) using exploratory factor analysis to highlight the practical application for training design, performance analysis, and talent identification.

## 2. Method

**Figure 1:**
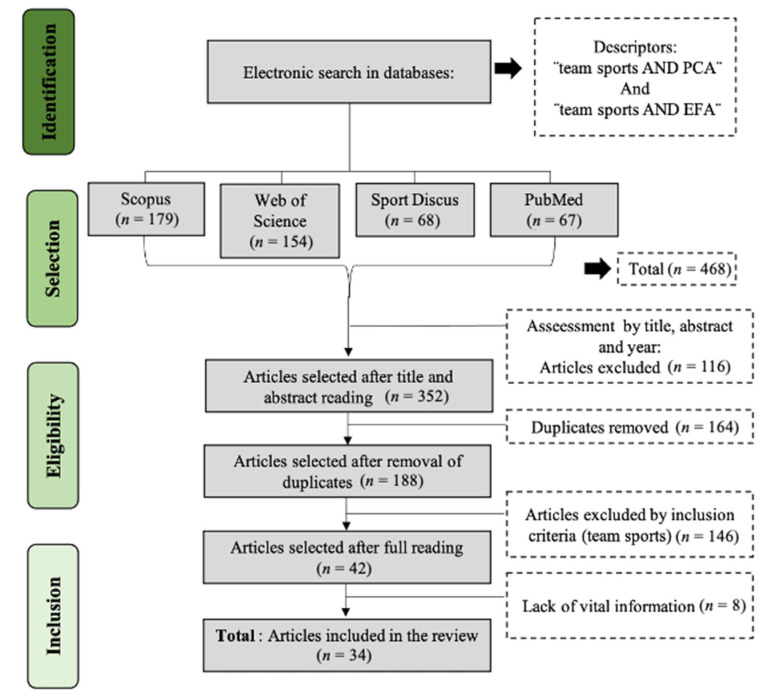
Flow diagram of the study.

A systematic review was performed following the Preferred Reporting Items for Systematic Reviews and Meta-Analyses (PRISMA) guidelines [19]. The procedure realized for data identification, selection, and extraction is presented in Figure 1.

### 2.1. Data Sources

A systematic electronic search was computed from PubMed (*n* = 67), Web of Science (*n* = 154), SPORTDiscus (*n* = 68) and Scopus (*n* = 179) on 1 November 2019 before 9:00 a.m., in order to identify studies that use the PCA in team sports as a data reduction technique. The authors were not blinded to journal names or manuscript authors. The search strategy combined terms covering the topics of the population (“team sport,” soccer, football, basketball, rugby) and intervention (PCA; “principal component analysis”, “exploratory factor analysis”). The search was made using combinations of the following terms linked with the Boolean operators “AND” (inter-group Boolean operator) and “OR” (intra-group Boolean operator, only for the second). Studies were included if PCA was made in the most-studied team sports (soccer, rugby, or basketball) following previous research [16].

### 2.2. Data Collection

One of the authors downloaded the primary data from the articles (title, authors, date, and database) to an Excel spreadsheet (Microsoft Excel, Microsoft, Redmond, WC, USA) and removed the duplicate records. Then, two authors screened the search results independently against inclusion/exclusion criteria. The references that could not be eliminated by title or abstract were retrieved and independently evaluated for inclusion. The authors were not masked to the title or authors of the publications. Any disagreements (2%, *n* = 11) on the final inclusion-exclusion status were resolved through discussion in both the screening and excluding phases, and the final decision was an agreement between authors. Abstract and conference papers from annual meetings were not included due to the lack of information needed to systematize (e.g., PC cumulative %, PCA total variance explained, eigenvalues, statistical and methodological crucial information). The additional information provided by the authors was considered during the screening process. Lack of other forthcoming details led to the article being excluded. Documents from all languages were included but were excluded if a translation may not be done.

### 2.3. Data Selection

Two authors performed the final studies’ selection and information extraction. The systematization of the data was made using methodological outcomes and results of the studies. The methodological approach was made analyzing the criteria used to perform exploratory analysis considering retention loading criteria, data suitability testing, extraction method used, factor and loading retention criteria selected, and rotation method if performed (see Table 1). The EFA outcomes were resumed considering author, year, sport (discipline), variables characteristics, participants information, sample size, number of extracted factors, percentage of variances explained, number of variables extracted, and final extraction outcomes (see Table 1).

## 3. Results

A total of 468 articles was initially retrieved from the mentioned databases, of which 116 were excluded considering the title, abstract and year of publication. After duplicate removal, a total of 188 articles was analyzed, contemplating exclusion and inclusion criteria. Finally, the full text of 42 studies was read and, due to a lack of vital information, eight studies were not considered. Therefore, 34 articles were included in this review (Figure 1).

### 3.1. Study Characteristics

Big data reduction through PCA was performed in 34 articles, clustered in different team sports: 17 in soccer, 11 in basketball, and 6 in rugby. The extracted variables belonged to five metrics: technical, tactical, biomechanical, physical/physiological and anthropometrics. Overall, the most considered metric was physical/physiological.

#### 3.1.1. Soccer

From the 17 articles on soccer (Table 1), four articles aim to identify the technical patterns that define players’ behavior, five articles to analyze the tactics, two articles to explore biomechanical aspects, ten articles to assess the physical/physiological requirements, and five articles to characterize the anthropometrical variables. Overall, from 54% to 81% of the total variance was explained through ≤23 variables.

#### 3.1.2. Basketball

From the 11 articles on basketball (Table 2), five articles extracted variables related to technical patterns, one article evaluated biomechanical aspects, five articles characterized physical/physiological requirements, and two articles assessed anthropometrical metrics. In general, the percentage of total variance explained was higher than 62% through 14 or fewer variables.

#### 3.1.3. Rugby

From the six articles included in the qualitative synthesis (Table 3), five articles studied the physical/physiological requirements, and one article analyzed the tactical variables. In general, the percentage of total variance explained from those variables that formed PC was between 52% and 90%, which was formed by 38 variables to explore tactical analysis and lower than 9 for physical/physiological requirements.

## 4. Discussion

The present systematic review sought to identify the most relevant variables to explain players’ performances, extracted through PCA in soccer, basketball and rugby to highlight the practical applications for training design, performance analysis and talent identification. In soccer, together with some anthropometric measures, relative age is the most important factor for talent identification. In the sport of soccer, a key tactic taught by coaches is occupying space during the game. In addition to controlling the ball, passing, and shots on goal, issues related to tactics and space occupation should guide training task design, ensuring that a high level of aerobic endurance in combination with very intensive and short actions is employed. In basketball, both anthropometrics and some physical capacity tests (sprint, flexibility, and agility) should be performed for talent identification. Like other team sports, training tasks in basketball should be based on a high level of aerobic endurance and the required acceleration/decelerations, but unlike other sports, landings impact, and body positioning gain relevance. While in rugby, as in other sports, high-intensity actions need to be trained, but for rugby this needs to be in combination with collisions. In addition, the tactics or style of play and high-intensity game pace are very relevant for this team sport.

A player’s external perception defines the motor behavior required to respond, which in turn determines the resultant motor action. These can be categorized into four main conditional dimensions each sport-specific action. Specifically, a player act using a motor skill (technical dimension) that requires a movement (conditional dimension), according to the player’s decision making (tactical dimension), and is conditioned by the player’s psychological state (psychological dimension) [49]. The uncertainty and non-linearity of the team sports games’ environmental nature lead to engaging these dimensions during the competition, without the possibility of pre-establishing what action will perform in each situation, making team sports unpredictable. In this context, it is why behavior analysis has become crucial to better understanding team sport athlete preparation.

The present systematic review, analyzed studies published in team sports that, applied the PCA technique. Practically, the importance of this systematic review may be considered in three ways: (1) team staff decision-making focusing training processes on the most relevant performance manifestations (high-intensity actions, lower intensity actions, short-explosive actions), (2) making more efficient training and competition analysis processes, which remains crucial due to the congested schedules in which many of the sports are usually involved, and (3) to highlight the most relevant variables for talent identification.

### 4.1. Soccer

One of the most relevant challenges for coaches of young soccer players is to develop training processes to determine a particular potential to become a professional player. Players’ anthropometrics (i.e., weight, height, biceps/triceps, subscapular, supra iliac measures) are essential performance indicators for talent identification. However, most studies identified that maturity is one of the most critical variables in this regard [25,26,32,34]. To date, the current research in soccer talent identification reports a systematic bias in selection towards players born early in the year (relative age effect) [18]. Those players with early maturity tended to have better physiological and technical performance. Subsequently, they are more influential on the game and recognized as more talented [18].

But, all dimensions of a team performance should be trained using soccer-specific situations. Therefore, technical and conditioning (in addition to psychological) dimensions should be developed during soccer-specific tasks in which tactical positioning should be the main basis [49]. In this regard, authors have highlighted the teams’ surface area (occupied space) as the main variable to assess team positioning [20,21,22]. This variable may be suitable for evaluating team behavior during different game phases and training the team to act in different game phases, where the attacking team should occupy a greater area than the defending team [50]. This fact is suitable because players perform in greater spaces per player and more inter-player distances during the attacking phase. In contrast, in the defending phase, players should maintain lower inter-player distances to close space within the convex hull, avoiding the attacking team’s progression.

However, team tactics are governed by inter-player connections through technical actions [51]. In this sense, training those tasks based on collective tactical positioning should be constrained to improve motor skills. Based on PCA, the main technical variables are the control of the ball, passes, and shots, specifically, in young soccer [25,32,34]. So, coaches should design training tasks with continuous role changes, ensuring players concentrate to coordinate sudden movements with teammates from greater areas to lower to improve positional decision making, in combination with a high number of ball controls, passes, and shots. This fact is consistent with Cordón-Carmona et al. [51], who explained that pass networks imply passes and controls are a vital game issue.

Finally, physical fitness and conditioning in soccer should be achieved using high-intensity intermittent actions (anaerobic endurance), with lower intensity and longer efforts (aerobic endurance), considerable power and strength aimed to perform very fast and intensive actions (neuromuscular efforts)—all of these together can results in agility and flexibility training tasks. Therefore, from the more than 200 variables that may be extracted from internal/external load, the challenge is to identify the most relevant variables in each sense. From the studies identified that perform principal components, 17 variables have shown the highest percentages explaining players performance: anaerobic endurance (i.e., angular velocity, speed displacements, distance at high metabolic load, HSR, sprint running, maximum velocity), aerobic endurance (i.e., distance covered, distance covered slow than 6 km/h, distance covered at between 21–24 km/h, metabolic power, dynamic stress load), and neuromuscular efforts (i.e., jumps, impacts, accelerations, decelerations, maximum acceleration, maximum decelerations).

In summary, both head and physical fitness and conditioning coaches should design training tasks in which players’ are required to have an optimal use of occupied space. Simultaneously, they need to perform many ball controls, passes and shots during high-intensity aerobic endurance, combined with a high number of impacts and accelerations/decelerations. For example, those task designed based on differential learning has been demonstrated as efficient to improve players performance from different dimensions (technical, tactical, and conditional) at the same time [52,53].

### 4.2. Basketball

Basketball as a sport has some unique qualities, which is different from other team sports, when considering how to assist coaches with athlete selection decision-making. Different authors have reported hand measures, height, weight, muscle mass and fat mass or body fat % are the main anthropometric factors important for talent identification in basketball. In fact, in talent identification programs, anthropometric measures have become one of the most important measures to consider, with it reported that anthropometrics metrics formed the second largest principle component in basketball talent identification, explaining the 20 % of the variance [54]. Moreover, the first and the third component related to talent identification is related to sprint test performance from 10 to 30 m, plus flexibility and agility variables. These metrics and variables are consistent with those identified in this systematic review, in which these tests are supported for talent identification processes, together with countermovement jump and squat jump testing [42]. Therefore, anthropometrical variables and sprint, flexibility and agility tests seem to be the primary basis for talent identification in basketball.

When considering the conditional dimension, unlike football, in basketball, both technical and conditioning dimensions have formed the main research topic of performing data reduction through PCA. In contrast, positional decision-making variables (tactical dimension) have not been widely investigated in basketball [55], and its application has been lower than in soccer [56,57,58]. For conditioning in basketball, distance over 18 km/h, max accelerations and decelerations, together with impacts of 3–5 g, average landing and take-off, and relative distance have been found as principal components between basketball’s load variables [15,43,44]. This is consistent with these studies highlighting that lower spaces per player are related to a high number of accelerations/decelerations and more high-intensity displacements [59,60]. When designing training tasks focused on these variables, free-throws, 2 and 3-points, passes, turnovers, defensive and offensive rebounds have been highlighted as essentials requirements [17,35,36,37].

Together with task focus on performance improvement, and the highlight that sudden basketball-specific action variables identified by the principle component (i.e., throws, turnovers, rebounds, acceleration/decelerations, and take off) make basketball a team sport with a high incidence of injury [61,62]. Thus, training processes should aim to, together with coadjutant exercises to focus on injury prevention. Specifically, injury prevention programs should be done during the in-season period because of their greater effectivity than pre-season, at least, in anterior cruciate ligament’s injury prevention [61]. Suggestions by Stojanovic and Ostojic [62], reported that stretching, proprioception, strength, plyometric and agility drills with additional verbal and visual feedback on proper landing technique lead to a decreased rate of an anterior cruciate ligament injury in team sport athletes. Due to the high eccentric contractions during basketball-specific actions, overuse and inflammatory conditions accounted for more than 39% of injuries during a 32 weeks basketball season [63] and, therefore, coadjutant training programs should consider this fact.

### 4.3. Rugby

From a holistic viewpoint, players’ and teams’ positioning is the main basis on which to develop the remaining dimensions (technical, conditioning, and psychological). Although distances between rugby players has been assessed [64], it is not widely evaluated [65]. One study reported that time of possession, speed of play and playing form variables are the appropriate measures to assess playing tactical issues. However, further studies are needed to extract robust conclusions [8]. Similar to the tactical dimension, the most relevant technical variables were not extracted. Therefore, although some actions such as collisions/tackle and passes are highly considered in rugby [66,67], PCA is necessary to extract the most critical information from technical actions.

The use of technology capable of extracting physical and physiological variables is widely used in rugby [66]. Therefore, they are the dimension most considered to extract the most relevant variables [13,45,46,48]. Rugby is a team sport in which players engage in a repeated high-intensity exercise involving frequent collisions, especially during the most demanding passages [66]. To assess players’ performance during matches, the most relevant variables were aerobic/anaerobic endurance (rating of perceived exertion, cumulative load, week to week load increasing, heart rate, player load and high-speed distance), in combination with high-intensity impacts [13,45,46,48]. Therefore, coaches should design training tasks in which the players perform a robust high-intensity activity (i.e., HSR and sprint) separated by short bouts of lower intensity activities (i.e., standing, walking, and jogging), together with collisions and wresting bouts [47,66]. This task could be based on worst-case scenarios to ensure players’ performance in these critical situations [66]. In these cases, high-speed running efforts and the importance of tackle success is important [67]. As a consequence of the high-frequency collisions, especially the head/face collisions, complementary training programs are essential to reduce injury incidence [68,69].

Overall, each team sport’s structural constraints define the players’ and teams’ behavior, and subsequently, main variables regarding talent identification, training design, and match analysis differ between them. However, team sports are similar in the fact that they are high-intensity sports in which the performance of high degree of aerobic endurance is mixed with sub-maximal strength and power aimed to carry out fast and intensive actions such as jumps, accelerations or decelerations. The implication of abilities, decision-making, movement and psychological state is implied in the players’ motion action, makes that multivariate analysis processed should be made to warrant optimal player performance in the most critical session: the official match.

However, in this systematic review, there was no work in which a data reduction technique was used to reduce the data set in which multivariate data variables were involved. Therefore, the most relevant variables of each dimension in each team sport were extracted, focusing only on a unique dimension. In this regard, general conclusions identify that a combination of 3–4 principle components is needed to explain team sports analysis performance, at least when extraction criteria were set at an eigenvalue of greater than one. Intensity training load metrics with principle component “loadings” above 0.6 or 0.7 were deemed to possess well-defined relationships with the extracted principle component.

## 5. Conclusions

Since team sports’ performance depends on different dimensions (i.e., technical, tactical, and conditional), multivariate data analysis should be performed in three ways: to make an efficient game analysis, design training tasks based on the most relevant efforts, and to highlight the most relevant variables for talent identification. The most critical variables extracted differ between team sports in each dimension were:

### 5.1. Soccer

- For talent identification: anthropometrical variables, together with a player’s relative age.
- For training design and performance analysis:
  ○ Tactical dimension: occupied space.
  ○ Technical dimension: ball control, passes and shot to goal.
  ○ Conditional dimension: angular velocity, speed displacements, distance at high metabolic load, HSR, sprint running, maximum velocity, distance covered, distance covered slow than 6 km/h, distance covered at between 21–24 km/h, metabolic power, dynamic stress load, jumps, impacts, accelerations, decelerations, maximum acceleration, maximum decelerations.

### 5.2. Basketball

- For talent identification: anthropometrical variables (hand measures, height, weight, muscle mass, and fat mass or body fat %), and 10 to 30 m sprint, flexibility, and agility tests seem to be the main basis for talent identification in basketball.
- For training design and performance analysis:
  ○ Technical dimension: free-throws, 2 and 3-points, passes, turnovers, defensive and offensive rebounds
  ○ Conditional dimension: distance over 18 km/h, max accelerations and decelerations, together with impacts 3–5 g, average landing and take-off, and relative distance

### 5.3. Rugby

- For talent identification: this review cannot give any information because of the lack of literature on this aspect.
- For training design and performance analysis:
  ○ Tactical dimension: possession, speed of play, playing form, and infringement.
  ○ Conditional dimension: rating of perceived exertion, cumulative load, week to week load increasing, heart rate, player load, high-speed distance, and impacts.

However, since these variables are extracted from different studies, further research should perform PCA from databases with variables from different dimensions and analyze the impact of different dimensions in the behavior of other dimensions (e.g., biomechanical in physical/physiological, tactic dynamics in technical efficacy).

## 6. Practical Applications for Training Task Design and Performance Analysis

Training tasks should ensure the development of players’ tactical, technical and conditional dimensions, both head and physical fitness and conditioning coaches should ensure training tasks used are only those in which all dimensions are considered. Based on different variables that formed each principal component in each team sport, training tasks in each sport should differ:

- Soccer: coaches should ensure that the occupied area is suitable during the game. Together with ball controls, passes, and shots, this fact should guide task design during a high level of aerobic endurance in combination with very intensive and short actions.
- Basketball: coaches should design training tasks based on high aerobic endurance level, together with a high number of jumps, landings, and impacts, in combination with passes and throws. However, future studies should assess what variables extract the most relevant information about players’ and teams’ tactical positioning through PCA.
- Rugby: coaches should design training tasks in which teams’ have to maintain a high number of possession time, together with a high velocity of game pace, while the players must perform high-intensity efforts in combination with collisions.

## Figures and Tables

**Table 1:** Variables extracted through principal component analysis (PCA) in soccer.

Ref.	Aim	Size	Nº of Initial Variables	N FACTORS	% Variance Explained	Nº Variables Extracted	Variables				
							Technical Dimension	Tactical Dimension	Conditional Dimension		Talent Identification
							Technical	Tactical	Biomechanical	Physic/Physiological	Anthropometrical
Moura et al. [20]	Players’ positional variability	Portugal, Spain, Italy, and Germany in EURO 2012	1	NR	NR	1		Ellipsis area 1st and 2nd component length			
Ric et al. [21]	Identify the tactical patterns and the timescales of positioning-derived variables that define the patterns during a match	20 male PRO	29	12	First half: 80.8% Second half: 80.91%	NR		Speed of dispersion, stretch index, length, width, sectors, corridors, longitudinal, and latitudinal team speed.			
Goncalves et al. [22]	Effects of the quality of opposition in spatial-temporal features that describe a team match	1 PRO team 12 matches, 1413 ball sequences	15	Team: ed5 Top: 5	Team: 72.2% Top: 74.2%	Team: 8 Top: 12		Ball position Team space occupation Space at the end of ball possession			
Torrents et al. [23]	Exploratory, technical, and tactical behavior in small-sided soccer games	PRO. 22 Amateur: 22	21	9–14	100%	NR		Attacker with ball. Attackers without ball Defenders.			
Carpita et al. [24]	Soccer statistics in actions of the play	Pro players	482	Home team: 3 Away team: 3	Home team: 75.8–73.2% Away team: 73.2–70.5%	Home team: 6 Away team: 11		Number of scoring opportunities, shots on goal, goal attack percentage, percentage of penalties, defensive headings, defensive actions, balls lost, etc.			
Abdullah et al. [25]	Prediction of technical skill relative performance	184 youth players	26	8	71.67%	16	Short pass Shooting RTC Shooting LTC			Sit up Agility 5–10–20 m speed	Weight Height Biceps/Triceps Subscapular, Suprailiac Maturity
Maliki et al. [26]	Develop Anthropometric, Growth and Maturity Index (AGaMI) in soccer	223 adolescent	26	2	76.37%	7					Bicep/Triceps Subscapular Suprailiac Maturation
Ricotti et al. [27]	Anthropometric, power, static and dynamic balance, contact time and reaction time	185 players (from amateur to PRO)	12	2	~70%	6			Force Static and dynamic balance with non-dominant limb and rapidity		
Ra et al. [28]	Identification of salivary fatigue markers in soccer players	122 male players	12	2	NR	NR				Pyruvate Acetyl-CoA Fumarate Succinyl-CoA Alanine Glycine Leucine Tryptophan Phenylalanine Tyrosine Isoleucine Valine	
Zago et al. [29]	Determinants in half-turn with the ball	10 U’13 players	34	7	79%	23				Angular velocity Distance and speed of displacements.	Center of mass Malleolus Pelvis and trunk range of movement
Abdullah et al. [30]	Identify the components of physical fitness related performance pattern	31 amateur players	8	2	54.06%	3				Maximum push-ups Vertical jump Maximum sit-ups	
Negra et al. [31]	Agility, speed, and power	95 male young	8	1	72.18%	8				Illinois agility test *t*-test 10-m and 20-m sprint Five jump test Countermove. jump Squat jump Abalakov	
Abdullah et al. [32]	Analysis of Essential Performance Indicators in Two Levels of Soccer Expertise	Elite young: 84 Novice: 100	27	Novice: 7 Elite: 7	Novice: 72.58% Elite: 69.51%	Novice: 10 Elite: 12	Ball control Long pass Short pass Shooting RTC shooting, LTC KMO			V Sit and Reach Sergeant jump Sit up variation Agility Speed 5 m Speed 10 m Speed 20 m VO2max	Age Weight Height Sitting height Bicep/Triceps Subscapular Suprailiac MUAC/CC Maturity
Los Arcos et al. [33]	Determine the underlying structure of the stretch-shortening cycle (SSC) jumping, acceleration, and change-of-direction (COD) abilities in soccer players	42 players	9	3	84.68%	9				5–10–15 m acceleration Free agility test 505 agility test 20-yd agility test Squat jump Arm swing Countermove. jump	
Maliki et al. [34]	Examine the most dominant variables multilaterally with specifically focused on the differences and inter-individually variability in different soccer positions	184 male youth players	33	9	66.62%	15	Ball shooting		Side post heading.	Speed Aerobic capacity Agility Flexibility	Weight Height Maturity SJ Biceps/Triceps Subscapular Iliac
Casamichana et al. [14]	Selection of external intensity training loads	24 PRO male players	10	Small Possession SSG: 3 Medium Possession SSG: 3 Small SSG: 3 Medium SSG: 3 Large SSG: 3 Off. match: 3	Small Possession SSG: 82.38% Medium Possession SSG: 78.17% Small SSG: 82.11% Medium SSG: 79.49% Large SSG: 80.50% Off. match: 85.62%	Small Possession SSG: 9 Medium Possession SSG: 9 Small SSG: 10 Medium SSG: 10 Large SSG: 10 Off. match: 10				Distance Dist. High meta. load HSR/Sprint running Max. velocity Acc/Dec Metabolic power Dynamic stress load Impacts	
Oliva-Lozano et al. [9]	Identify the representative external load profile of match-play	26 male PRO players	49	4	66.8%	11				Distance Dist. 0–6; 21–24 km/h Distance HSR Acc HSR Sprints Acc and mean Acc Max Acc/Dec Max. Velocity	

PCA = Principal Components Analysis, NR = Not Reported.

**Table 2:** Variables extracted through PCA in basketball.

Ref.	Aim	Size	Nº of Initial Variables	N Factors	% Variance Explained	Nº Variables Extracted	Variables				
							Technical Dimension	Tactical Dimension	Conditional Dimension		Talent Identification
							Technical	Tactical	Biomechanical	Physic/Physiological	Anthropometrical
Sampaio et al. [17]	Effect of contextual variables in game-related statistics	306 games of PRO Spanish league	14	5	82%	7	Free-throws 2 and 3 point, 3-point passes Errors Def/Off rebounds				
Liu [35]	Simplifies each indicator that affects basketball team integrated technical score	23 games	17	5	79.67%	11	Shots 2-point shot 3-point shoot average Free throws Def/Off rebounds Assists Steals Turnovers, Fouls Score				
Yin [36]	Technical and efficacy index	10 elite-level NBA players	10	3	89.48%	10	Playing time Score Steal Nº of faults Games played Rebound Block shot Assist Field-goal % free-throw %				
Yin [37]	Gets players’ ability comprehensive indicator model.	97 CBA foreign players	10	3	89.48%	6	Playing time Score Steal Nª of faults Games played Rebound				
Andrade et al. [38]	Biomechanics of jump	19 female PRO	9	Static jump: 2 Jump with approx: 3	Static jump: 73.39% Jump with approx.: 79.15%	Static jump: 4 Jump with approx.: 5			Passive force peak Time to reach the passive and propulsive force peak Mean speed Load Concent/Eccent. phase duration		
Svilar, Castellano and Jukic [39]	Positional differences: Selecting appropriate training-load measures	13 PRO players	10	Guards: 3 Forwards: 3 Centers: 2	Guards: 100% Forward: 100% Centers: 92.96%	Guards: 6 Forwards: 7 Centers: 8				Total/high Acc/Dec Total/high COD Total/high jumps RPE	
Teramoto et al. [40]	Predictive validity of the NBA Draft Combine on the future performance of basketball players	1092 NBA draft players	23	3	72.4%	12				Vertical jump Max. jump 3/4 court sprint, Lane agility Bench press	Wingspan Hand length/width Height without Shoes Standing reach Weight Body fat %
Floría et al. [41]	Force, velocity, and displacement-time curves	34 trained women	3	Force: 6 Velocity: 4 Displacement: 2	97 ± 0.35%	3				Force Mean propulsive velocity Displacement-time curves	
Hilgemberg et al. [42]	Anthropometric, power, agility, speed, and endurance	22 national-level youth	13	3	79.7%	11				Squat jump Countermove jump Abalakov Agility test 10 and 30 m sprint Abdominal endurance Medicine ball throw Oxygen uptake	Height Weight Fat mass Muscle mass
Pino-Ortega et al. [43]	Set kinematic behavior parameters during official matches	94 young elite	252	3	66.3%	6				Peak/Average Acc Landing 8–100 Rel. Distance Jump Av. Take-off/landing	
Rojas-Valverde et al. [44]	Identify the external workload representative variables	12 elite basketball referees	44	3–5	74.7–80.6%	10–14				Dist. 0–6, 18–21, 24–50 km/h Max. Acc. Max. Speed Impacts 3–5 g	

PCA = Principal Components Analysis, NR = Not Reported.

**Table 3:** Variables extracted through PCA in rugby.

Ref.	Aim	Size	Nº of Initial Variables	N Factors	% Variance Explained	Nº Variables Extracted	Variables				
							Technical Dimension	Tactical Dimension		Conditional Dimension	Talent Identification
							Technical	Tactical	Biomechanical	Physic/Physiological	Anthropometrical
Parmar et al. [8]	Selecting appropriate measures	45 players	45	10	81.8%	38		Possession Speed of play Form Infringement variables			
Weaving et al. [13]	Internal and External Training Load measures	17 PROmale	5	SSG/SSCG: 1 Skills, wrestle, strongman, and speed: 2	SSG: 68% SSCG: 52% Skills: 68% Wrestle: 71% Strongman: 72% Speed: 67%	5				Ind. TRIMP RPE Body Load Dist. HSR Total impacts	
Williams et al. [45]	Variable selection of training-load measures	173 PRO rugby union players	10	3	90%	9				Daily TL (1–2–3–4) cumulative load Week-to-week change Training strain Acute: chronic workload ratio Exponentially weighted moving average	
Weaving et al. [46]	The effect of training mode on multivariate training load	23 PRO male rugby players	4	Skills: 1 Conditioning: 2	Skills: 57% Conditioning: 85%	Skills: 2 Conditioning: 4				RPE HR Dist. HSR Player Load	
Henderson et al. [47]	Physical and technical match performance	20 PRO rugby 7’s players	18	Physical performance: 4 Technical performance: 2	Physical performance: 62.30% Technical performance: 88.85%	Physical performance: 4 Technical performance: 2				Speed HSR/min VHSR/min Max. speed Total carries Total passes	
Weaving et al. [48]	Identify which combination of external and internal TL metrics capture similar or unique information for individual players	21 male PRO players	4	2	89.6%	4				Distance Player Load Dist. HSR RPE	

PCA = Principal Components Analysis, NR = Not Reported.
